# A Simplified Technique for the Fabrication of Esthetic and Functional Space Maintainers in Pediatric Dentistry

**DOI:** 10.1111/jerd.70129

**Published:** 2026-02-22

**Authors:** Luciana Duarte Caldas, Anne Vitória de Jesus Ramos, Luísa Schubach da Costa Barreto, Katharina Morant Holanda de Oliveira Vanderlei

**Affiliations:** ^1^ Dentistry Department State University of Paraíba (UEPB) Campina Grande Paraíba Brazil; ^2^ Private Practice Ribeira do Pombal Bahia Brazil; ^3^ Social and Preventive Dentistry (PRECOM) Department Rio de Janeiro State University (UERJ) Rio de Janeiro RJ Brazil; ^4^ Dentistry Department Federal University of Sergipe (UFS) Lagarto Sergipe Brazil

**Keywords:** deciduous tooth, mixed dentition, orthodontic space maintenance, pediatric dentistry, preventive orthodontics

## Abstract

**Introduction:**

Premature loss of primary molars remains a frequent clinical problem in pediatric dentistry and may lead to space loss, occlusal disturbances, and the need for future orthodontic intervention. Conventional space maintainers, such as the band‐and‐loop appliance, are effective in preserving arch length but do not restore occlusal function or esthetics.

**Objectives:**

This case report describes an easy and practical technique for fabricating esthetic‐functional space maintainers without the need for silver or laser soldering during the laboratory phase, eliminates the need for orthodontic wire and without the use of CAD‐CAM technology.

**Clinical Technique:**

A 4‐year‐old child presented with extensive carious destruction, irregular distal root resorption and furcation involvement of the mandibular right first primary molar, for which extraction was indicated. Radiographic evaluation revealed that the permanent successor was at an early stage of development, justifying the need for space maintenance. A fixed esthetic‐functional space maintainer was fabricated using the anatomy of the child's own primary tooth as a guide. Clinical and radiographic follow‐up at 12 months demonstrated satisfactory space preservation and stable occlusion.

**Conclusions:**

This simplified, low‐cost technique represents a practical alternative for pediatric dentists seeking to restore function and esthetics while maintaining space during the mixed dentition period.

## 1 Introduction

The preservation of primary teeth plays a fundamental role in the development of the stomatognathic system, contributing to the establishment of normal occlusal relationships, guiding the eruption of permanent successors, and maintaining arch integrity during craniofacial growth [1, 2]. In addition to their functional importance in mastication and speech, primary teeth act as natural space maintainers, preserving the mesiodistal dimensions required for the proper alignment of the permanent dentition [3]. Disturbances during this critical period may result in irreversible occlusal alterations and increased need for complex orthodontic interventions later in life [4].

Premature loss of primary molars, particularly due to extensive caries, trauma, or furcation involvement, remains highly prevalent worldwide and represents a major risk factor for space loss, crowding, and the development of malocclusions during the mixed dentition [5]. Reported prevalence rates of early primary tooth loss range from approximately 18% to 35% [6], highlighting its relevance as a public health concern and reinforcing the importance of preventive and interceptive orthodontic strategies.

Space maintainers are routinely indicated when a primary tooth is lost at least 1 year before the expected eruption of its permanent successor or when the successor is still at an early stage of dental development [1]. Among the available options, the conventional band‐and‐loop appliance remains the most widely used for unilateral loss of a primary molar [7], mainly due to its simplicity, low cost, and ability to preserve arch length. However, this appliance is essentially non‐functional, as it does not restore occlusal contact, which may allow extrusion of the antagonist tooth, compromise mastication, and negatively affect vertical dimension stability [8].

To overcome these limitations, several functional modifications of the band‐and‐loop appliance have been proposed, aiming to restore occlusal contact and improve esthetics [8, 9, 10, 11, 12]. Although these designs have shown satisfactory clinical outcomes, most require orthodontic wire bending and silver or laser soldering, which increase laboratory complexity, technique sensitivity, and overall cost. More recently, digital workflows using CAD‐CAM systems and 3D‐printing technologies have been introduced for the fabrication of esthetic and functional space maintainers [8]. Despite their promising results, these approaches remain inaccessible to many clinicians due to the high cost of equipment, need for specialized training, and limited availability in public health and academic settings.

In this context, there is a growing demand for simplified, low‐cost, and reproducible techniques that allow the fabrication of esthetic‐functional space maintainers using conventional materials and analog workflows, without compromising clinical performance [4]. From a biological and developmental perspective, an often‐overlooked aspect of space maintenance is the preservation of physiological primate spaces, which play a crucial role in accommodating the eruption of permanent incisors and canines and facilitating spontaneous anterior alignment [2]. Few studies have specifically addressed whether commonly used space maintainers respect these natural spacing patterns or inadvertently eliminate them due to rigid design characteristics [1, 3, 6].

Therefore, the present report aims to describe an easy and practical technique for fabricating an esthetic‐functional space maintainer that restores occlusal contact, preserves arch length, and respects physiological spacing, without the need for orthodontic wire bending, silver soldering, laser welding, or CAD‐CAM technology.

## 2 Clinical Technique

**FIGURE 1:**
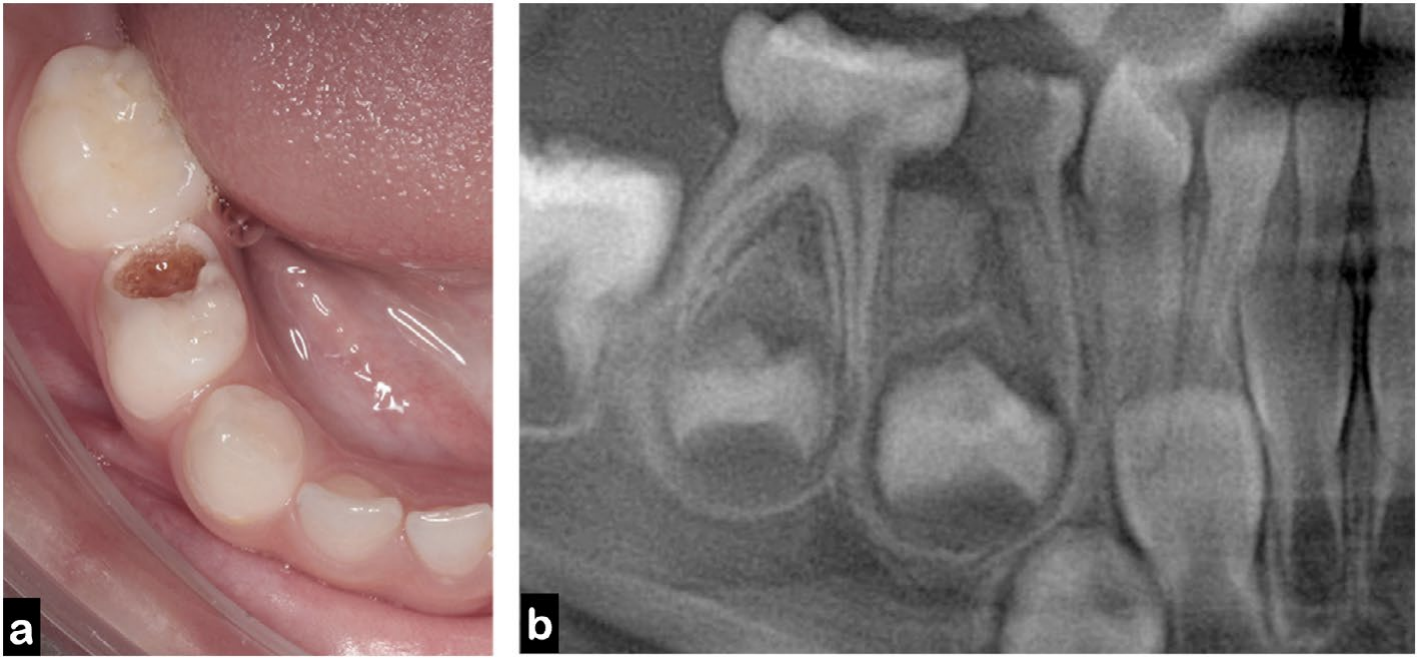
Initial clinical and radiographic findings: (a) intraoral lateral view showing extensive carious destruction of tooth 84 with furcation involvement; (b) periapical radiograph confirming irregular distal root resorption and early developmental stage of the permanent successor (tooth 44), classified as Nolla stage 5.

A 4‐year‐old child presented to the pediatric dentistry specialization clinic with a high caries risk, exhibiting multiple white spot lesions in the cervical regions of most teeth. Clinical examination revealed extensive carious lesions affecting the primary maxillary right lateral incisor (tooth 52) and the mandibular right first primary molar (tooth 84) (Figure 1a). The latter showed furcation involvement and irregular distal root resorption, which were confirmed by radiographic examination (Figure 1b). Given the severity of the lesion and the unfavorable prognosis, endodontic treatment was no longer indicated, and extraction was considered the only viable treatment option. At the time of diagnosis, the permanent successor (tooth 44) was at Nolla stage 5 [13] and Demirjian stage C [14] indicating an early stage of development and, therefore, justifying the indication for a space maintainer following extraction.

Written informed consent was obtained from the child's legal guardians for both the treatment and the publication of clinical data and images. In accordance with local regulations and institutional guidelines, approval from an ethics committee was not required for the reporting of a single clinical case.

**FIGURE 2:**
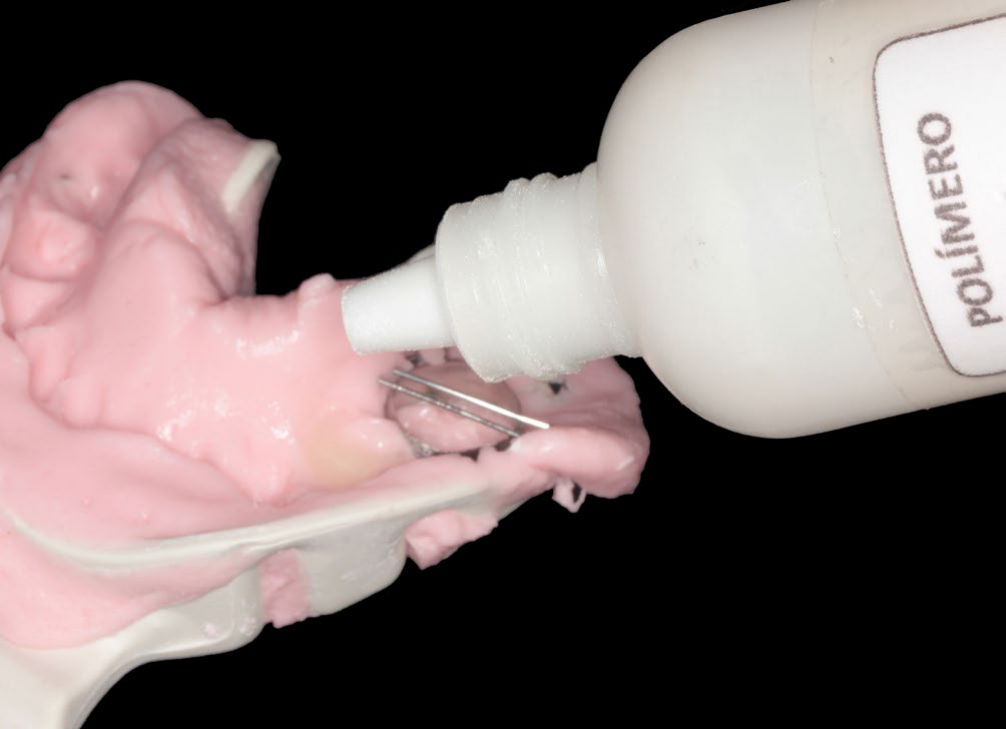
Transfer impression after orthodontic band selection on tooth 85 and prior to extraction of tooth 84. The area corresponding to tooth 84 was filled with tooth‐colored acrylic polymer.

**FIGURE 3:**
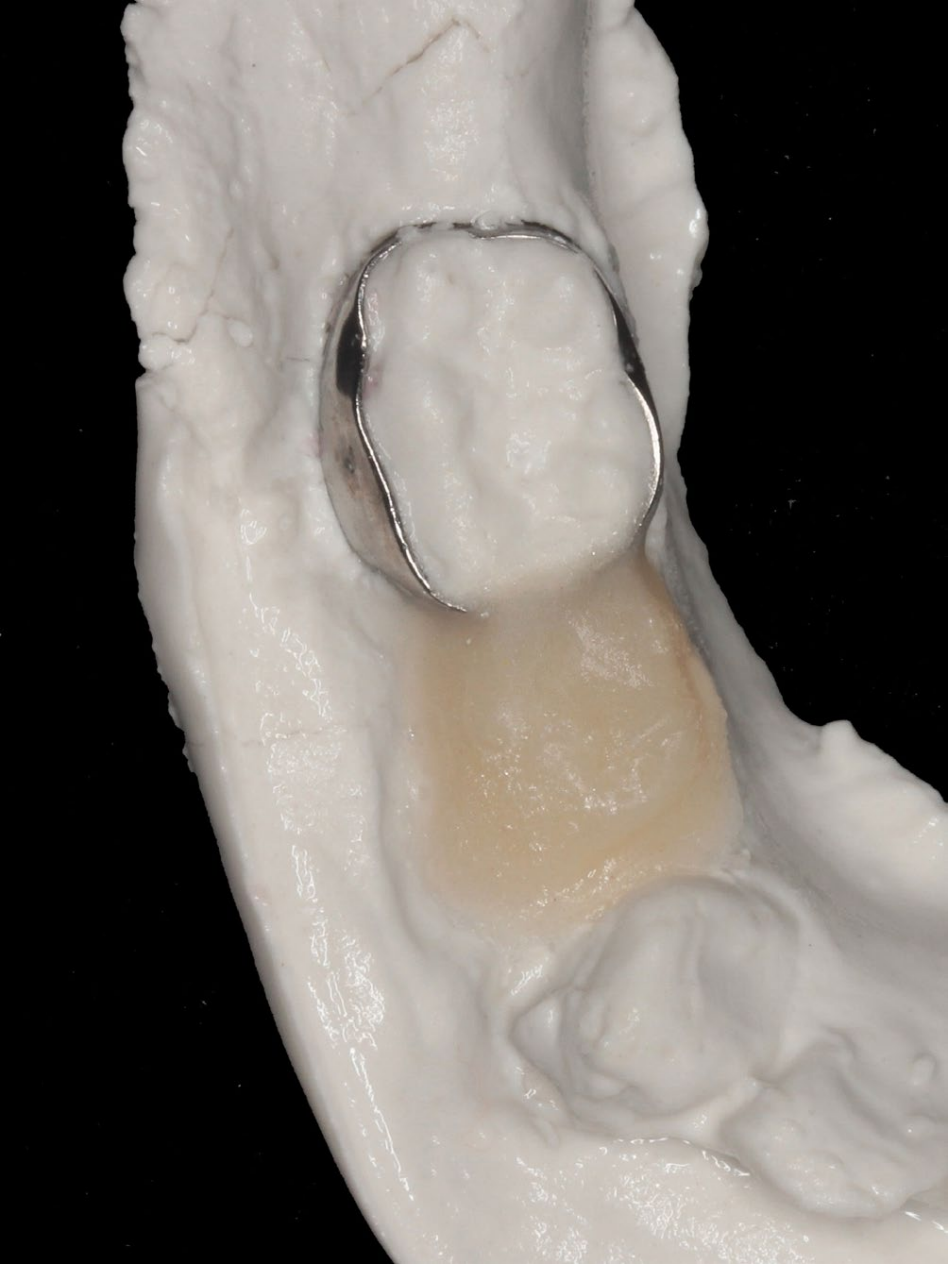
Working model obtained after pouring the impression with dental stone.

Prior to extraction, an orthodontic band was selected and adapted to tooth 85. A transfer impression was taken to allow fabrication of the appliance while preserving the original anatomical relationships. The region corresponding to tooth 84 was initially poured with a tooth‐colored acrylic polymer (Clássico, São Paulo, SP, Brazil) (Figure 2). After polymerization, the remaining portion of the impression was poured with dental stone (Pasom, Mairiporã, SP, Brazil) to obtain the working model (Figure 3).

**FIGURE 4:**
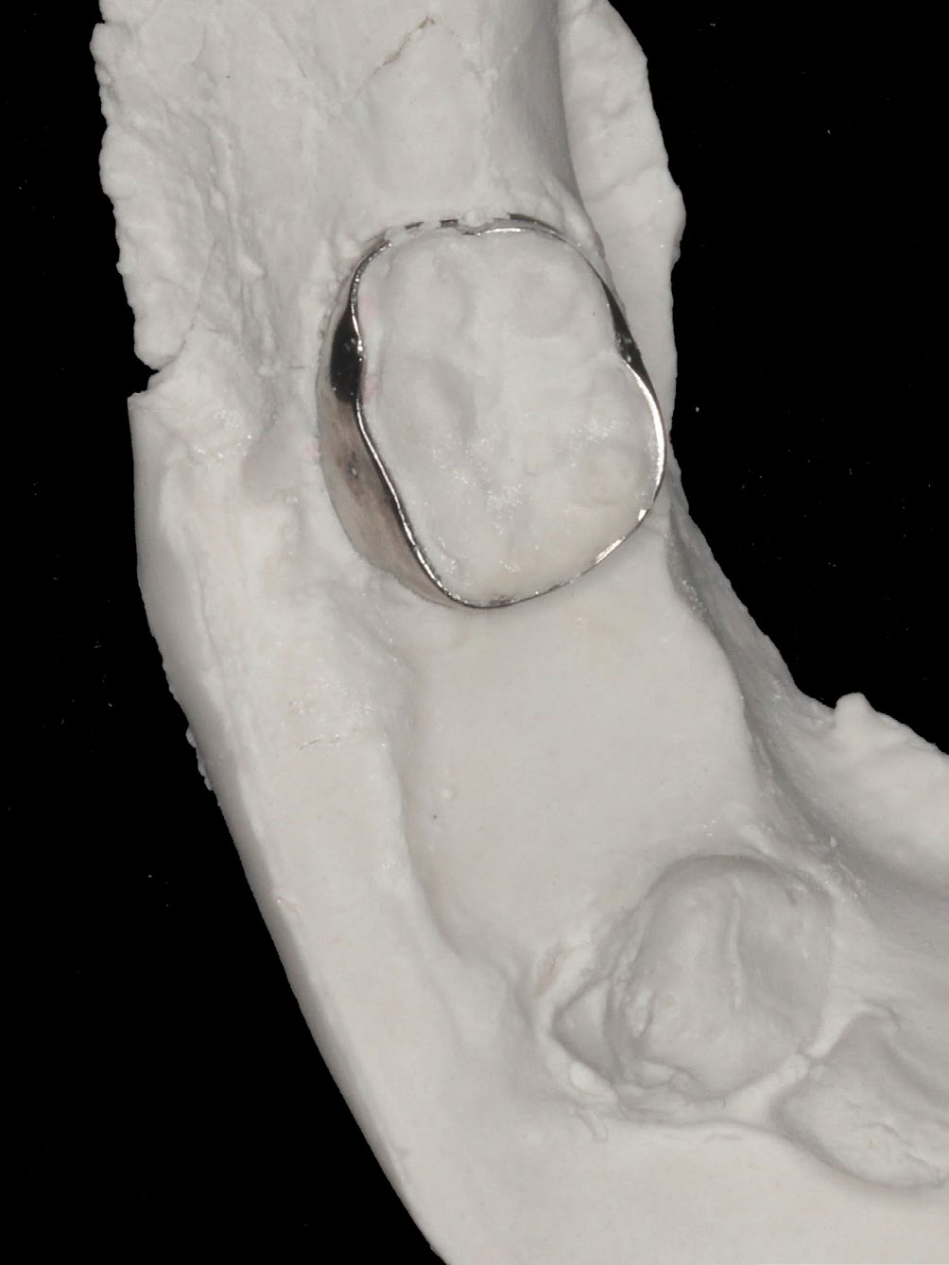
Removal of the acrylic prototype corresponding to tooth 84 from the working model.

**FIGURE 5:**
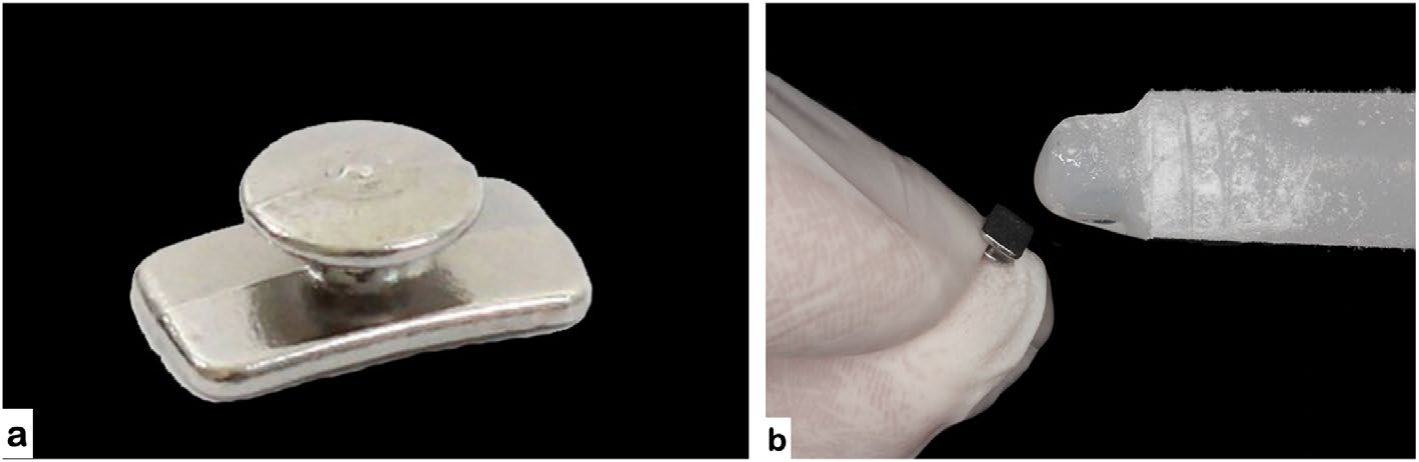
Orthodontic button used for appliance fabrication: (a) button prior to bonding; (b) application of ethyl cyanoacrylate adhesive.

**FIGURE 6:**
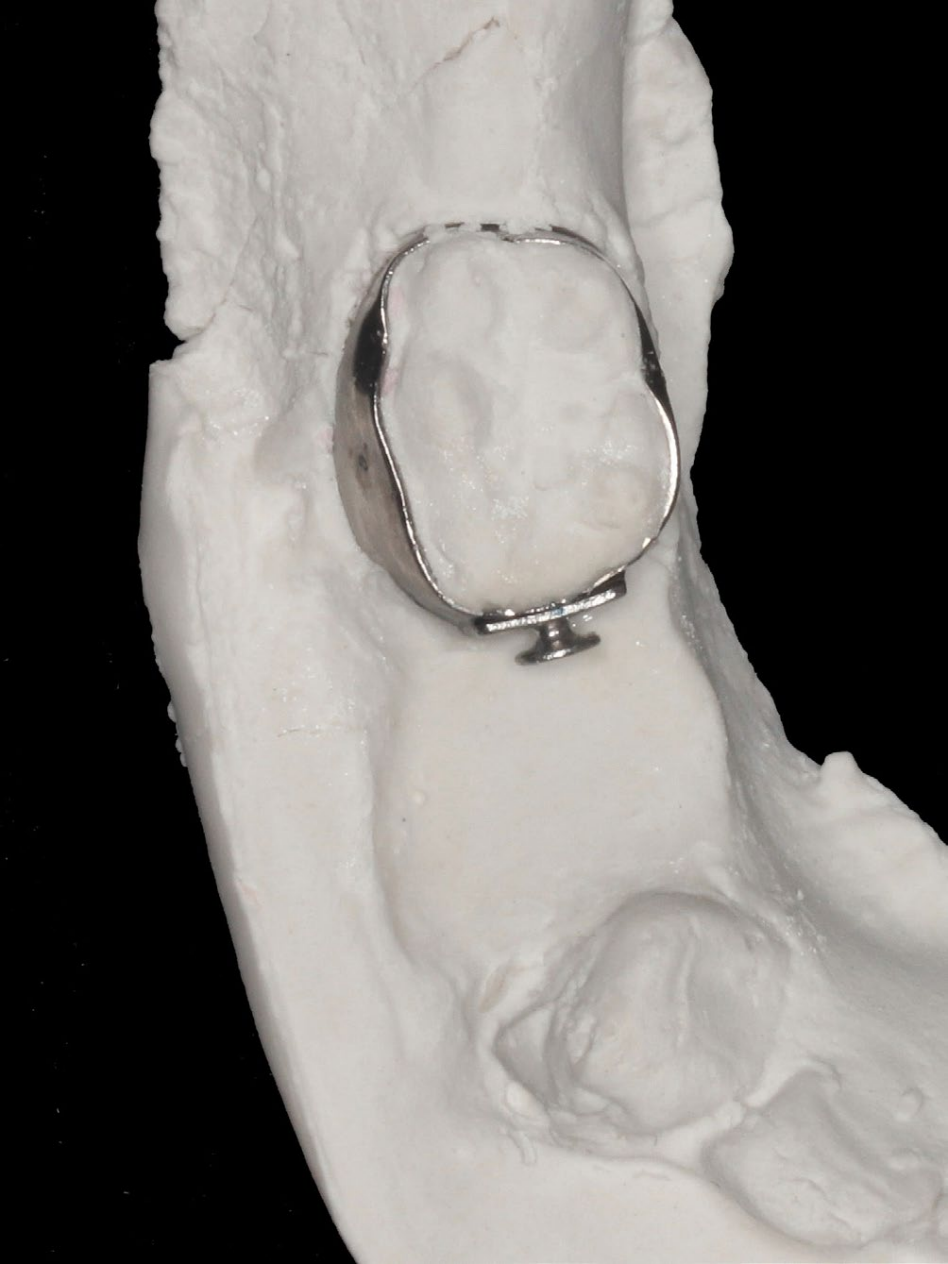
Bonding of the orthodontic button to the mesial surface of the orthodontic band.

The acrylic prototype of tooth 84 was removed from the model (Figure 4), and an orthodontic button (Morelli, Sorocaba, SP, Brazil) (Figure 5a) was bonded to the mesial surface of the orthodontic band using ethyl cyanoacrylate adhesive (Super Bonder, Hartford, CT, USA) (Figure 5b). Subsequently, the orthodontic band with the button was adapted and stabilized, ensuring proper fit and retention (Figure 6). After completion of the space maintainer fabrication, the appliance underwent full finishing and polishing and was installed in the oral cavity 30 days after the extraction of tooth 84, according to the treatment planning, since the clinical sessions of the specialization course were held monthly. However, the installation can and should be performed as soon as possible, respecting the healing time of the surgical wound for each patient in order to prevent any loss of arch length in the affected area.

**FIGURE 7:**
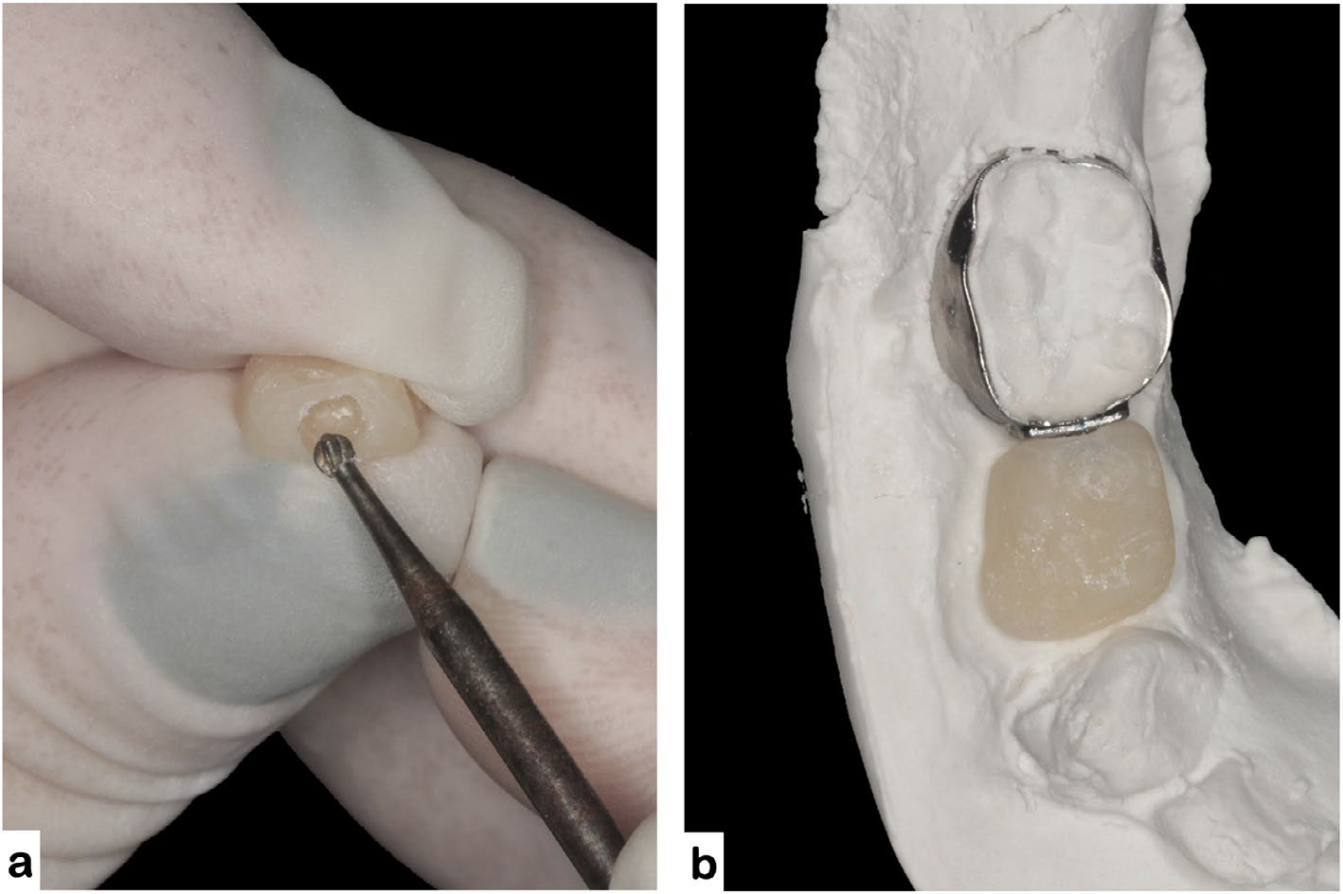
Finishing procedures of the acrylic prototype: (a) preparation of a distal retention hole using a carbide bur; (b) repositioning of the prototype on the working model to confirm anatomical alignment.

**FIGURE 8:**
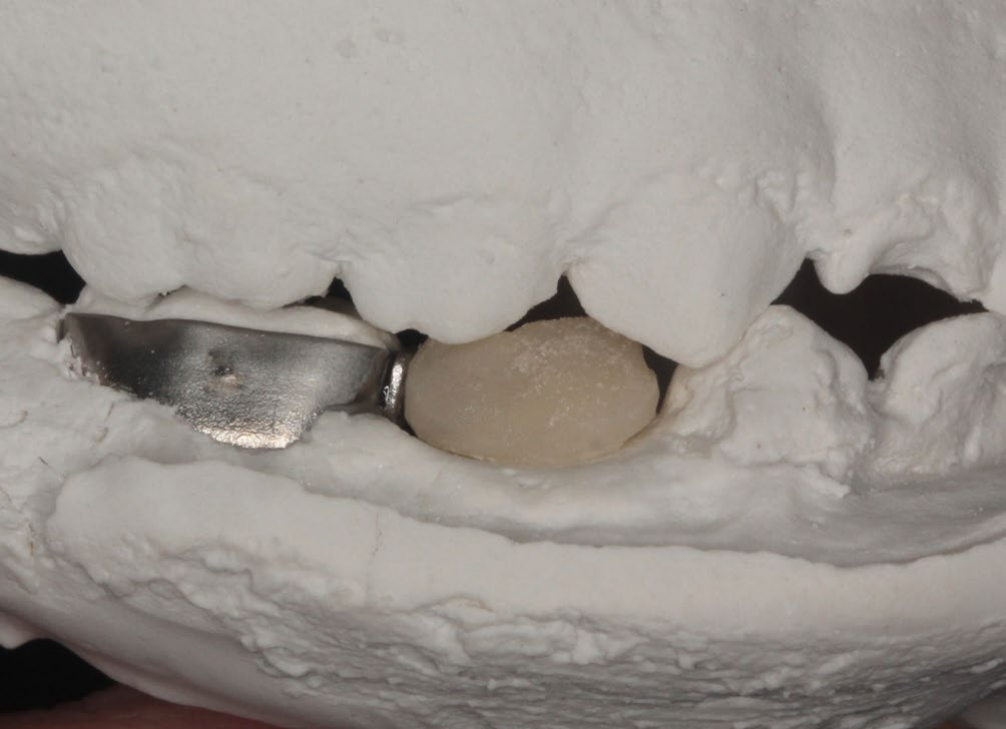
Maxillary cast positioned against the mandibular model to verify occlusal contacts and maintenance of vertical dimension.

**FIGURE 9:**
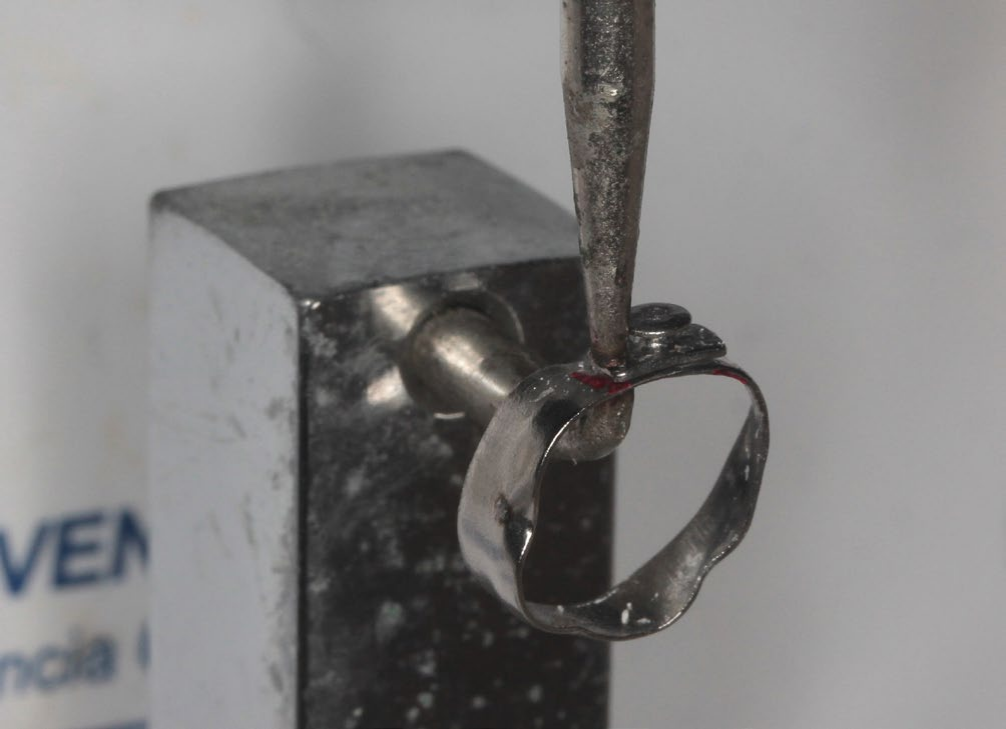
Spot welding performed to reinforce the metal union and improve mechanical stability.

After finishing and polishing, a small hole was created on the distal surface of the acrylic prototype using a carbide bur (Figure 7a), and the prototype was repositioned on the model to ensure proper anatomical alignment (Figure 7b). An impression of the maxillary arch was also obtained to allow verification of the vertical dimension and occlusal contacts (Figure 8). Spot welding was performed to reinforce the metal union and improve mechanical stability (Figure 9).

**FIGURE 10:**
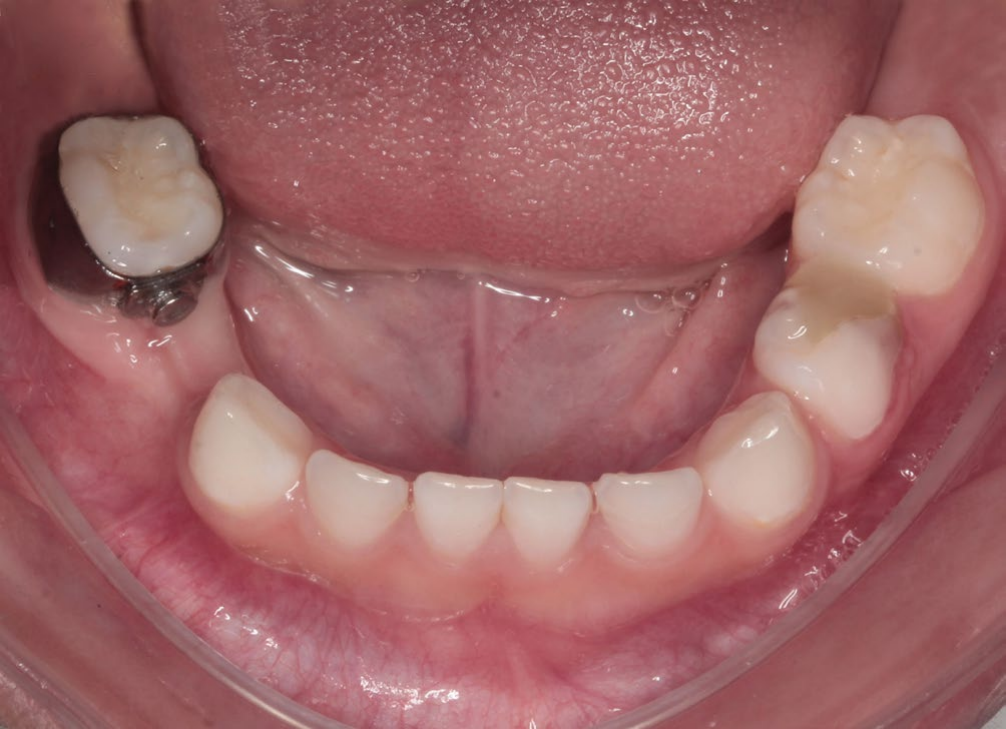
Cementation of the orthodontic band using self‐curing glass ionomer cement.

**FIGURE 11:**
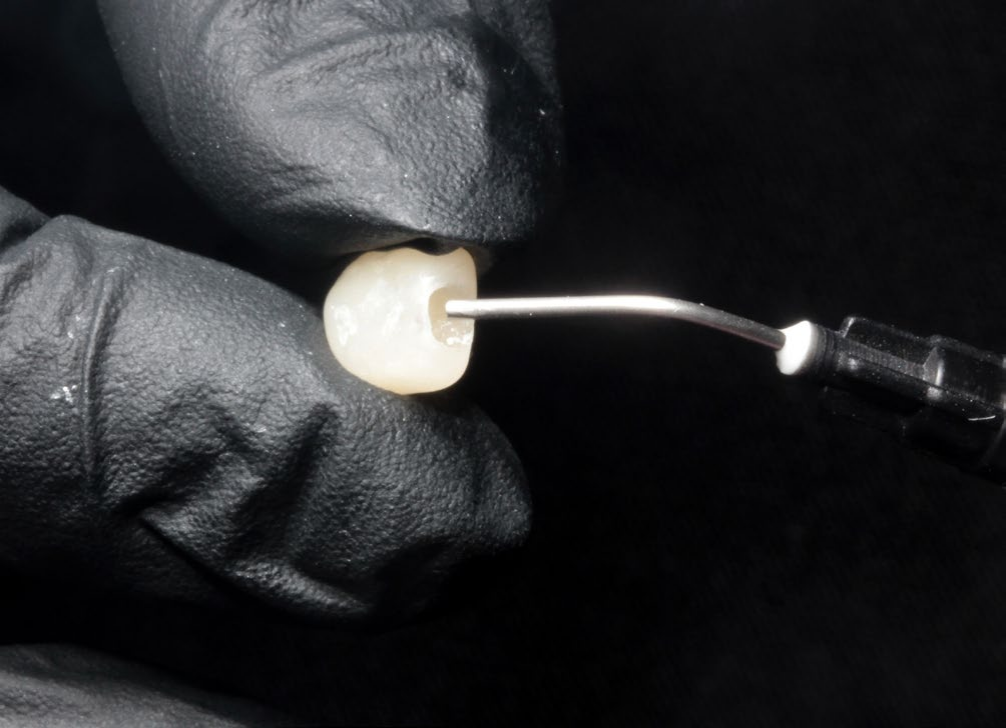
Filling of the distal retention hole with high‐viscosity light‐cured flowable composite resin.

**FIGURE 12:**
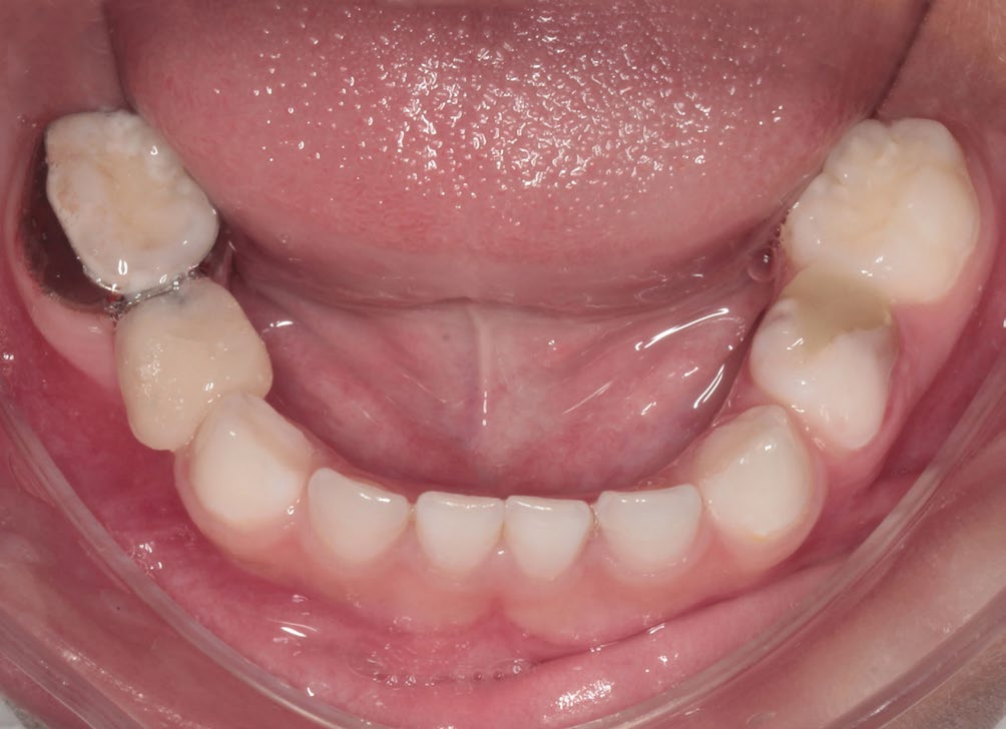
Attachment of the acrylic prototype to the orthodontic button after polymerization of the composite resin.

**FIGURE 13:**
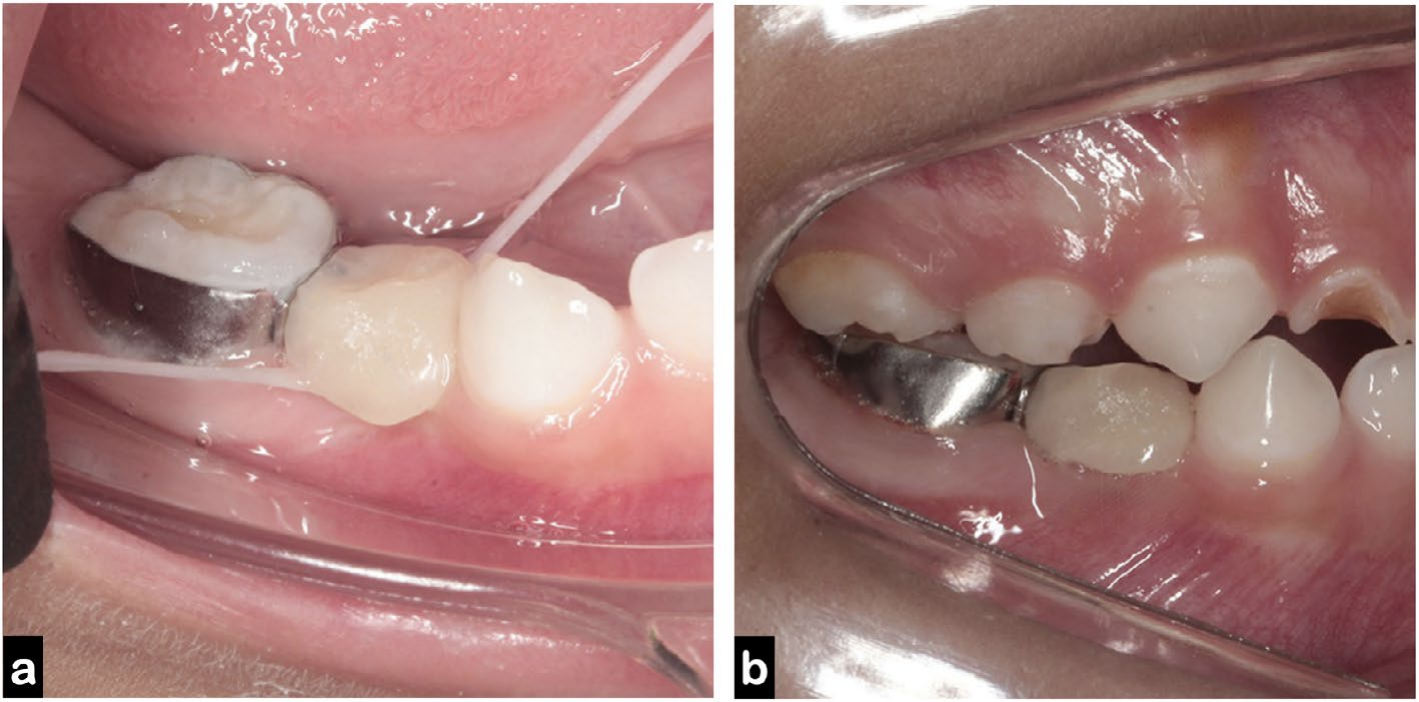
Clinical evaluation and instructions: (a) demonstration of oral hygiene and flossing technique adapted for the appliance; (b) verification of occlusal contacts after appliance installation.

The orthodontic band was cemented using a self‐curing glass ionomer cement (Meron, Cuxhaven, CUX, Germany) (Figure 10). The hole in the acrylic prototype was filled with a high‐viscosity, light‐cured flowable composite (Voco, Cuxhaven, CUX, Germany) (Figure 11), which was adapted onto the orthodontic button and polymerized (Figure 12). Oral hygiene instructions, including flossing techniques adapted for the appliance, were provided to the patient's caregivers (Figure 13a). Occlusion was carefully checked and adjusted when necessary (Figure 13b).

**FIGURE 14:**
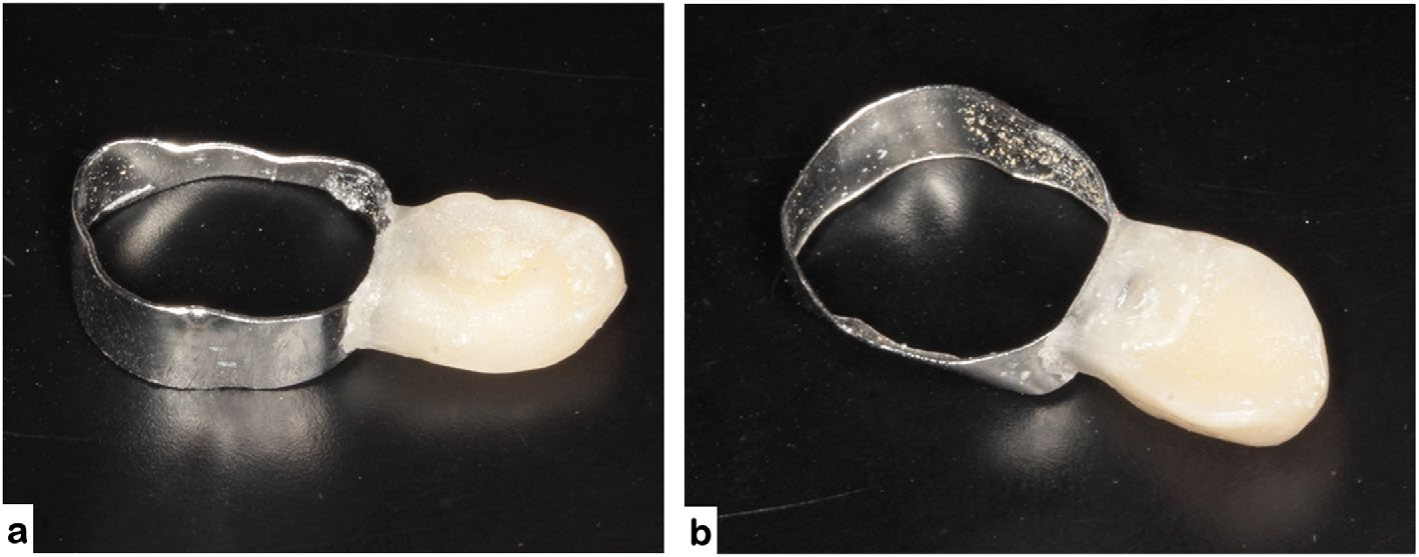
Alternative fixation method using acrylic resin: (a) occlusal view; (b) lateral view of the prototype attached to the orthodontic button.

As an alternative to flowable composite resin, the acrylic prototype can be bonded to the orthodontic button using a conventional monomer–polymer acrylic resin technique (Figure 14). The inner surface of the prototype is slightly roughened to enhance mechanical retention, followed by the application of monomer and incremental addition of polymer powder until a workable consistency is achieved. The prototype is then positioned onto the orthodontic button, ensuring proper alignment and intimate contact. After polymerization, the assembly is finished and polished to obtain a smooth surface and reduce plaque accumulation. This method provides adequate mechanical stability and may be useful in clinical settings where light‐cured composite resins are not readily available.

**FIGURE 15:**
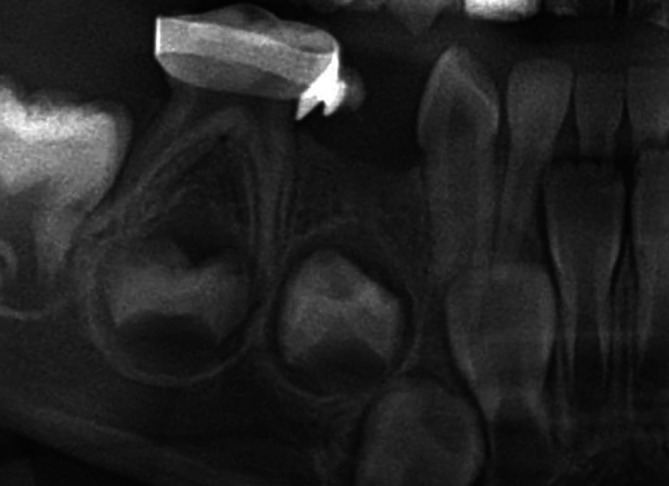
Radiographic follow‐up at 12 months showing preservation of the extraction space and normal development of the permanent successor.

At the 12‐month radiographic follow‐up, normal root development of the permanent successor (tooth 44) was observed, with adequate preservation of the extraction space and stable occlusal relationships (Figure 15). However, periodic annual clinical and radiographic follow‐up is essential to monitor the eruption of the permanent successor, evaluate the integrity and stability of the space maintainer, and ensure the preservation of proper occlusal relationships and masticatory function. Finally, after the clinical management of tooth 84, treatment was continued for the remaining dental units that required intervention, ensuring comprehensive oral rehabilitation and ongoing maintenance of the patient's overall oral health.

### 2.1 Follow‐Up and Outcomes

Space preservation was clinically assessed by verifying the maintenance of mesiodistal width between adjacent teeth and radiographically by monitoring the position and development of the permanent successor over time. Occlusal stability was evaluated through clinical examination of occlusal contacts, absence of extrusion of the antagonist tooth, and preservation of vertical dimension, using maxillary and mandibular casts to confirm functional intercuspation.

Clinical and radiographic follow‐up at 12 months demonstrated satisfactory maintenance of the extraction space, stable occlusal contacts, and normal eruption trajectory of the permanent successor, supporting its clinical effectiveness as a functional alternative to conventional band‐and‐loop appliances. No adverse effects, such as soft tissue irritation, appliance fracture, cement failure, or undesirable tooth movement, were observed during the observation period.

From a clinical perspective, the appliance showed good patient tolerance, satisfactory esthetic integration, and adequate functional performance. Given the clinical realities faced by pediatric dentists and orthodontists, particularly in low‐resource or high‐demand settings, this technique represents a practical, easy‐to‐implement, and low‐cost alternative. By eliminating the need for laboratory soldering or digital workflows, it simplifies fabrication while providing both esthetic and functional benefits.

## 3 Discussion

The present technique describes the fabrication of an esthetic and functional space maintainer through a simplified, fully analog workflow that reproduces the original anatomy of the primary tooth, without the need for orthodontic wire bending, silver or laser soldering, or CAD–CAM technology. In contrast to previously reported esthetic space maintainer approaches that depend on complex laboratory procedures, digital workflows, or rigid metallic components, this method enables chairside planning and laboratory execution using conventional materials. Consequently, it is particularly suitable for pediatric dental practice, educational settings, and public health services. The technique is indicated for cases of premature loss of primary molars in which the permanent successor is at an early stage of development and space maintenance with restoration of occlusal contact and esthetics is required, especially in clinical environments with limited access to advanced orthodontic or digital resources.

Premature loss of primary molars, particularly in the mandibular arch, has been consistently associated with rapid reduction of arch length, with most space loss occurring within the first 6 months following extraction [15]. Although conventional band‐and‐loop appliances are effective in preserving mesiodistal space, their inability to restore occlusal contact and esthetics has motivated the development of alternative functional designs. This early and pronounced dimensional change reinforces the importance of timely intervention, especially during critical periods of craniofacial growth [5]. In young children, fixed space maintainers are often preferred over removable appliances due to limited compliance, greater stability, and more predictable clinical outcomes [16].

The conventional band‐and‐loop appliance remains the most widely adopted option for unilateral space maintenance because of its simplicity, low cost, and effectiveness in preserving mesiodistal space [11]. However, its non‐functional design represents a well‐recognized limitation [4, 6]. By failing to reestablish occlusal contact, this appliance may allow extrusion of the opposing tooth, compromise masticatory efficiency, and potentially affect the vertical dimension [9]. Functional designs have therefore been proposed to address these shortcomings, aiming to restore occlusal relationships and improve oral function [10, 17]. The technique described in this report aligns with this functional philosophy while preserving the simplicity and affordability that characterize traditional space maintainers.

One of the main advantages of the present approach is the use of the child's own primary tooth anatomy as a reference for fabrication. This strategy enables preservation of natural contours, occlusal morphology, and mesiodistal dimensions, which are critical parameters during the mixed dentition phase. Even minor alterations in tooth shape or spacing may influence eruption paths, intercuspation patterns, and the development of occlusal relationships. By replicating the original anatomical form, the proposed technique may contribute to a more physiological integration of the appliance into the developing occlusion.

Another relevant aspect is the elimination of orthodontic wire bending and silver or laser soldering, which are commonly required in modified functional band‐and‐loop designs. These laboratory steps increase technical sensitivity, fabrication time, and overall cost, potentially limiting access to treatment, particularly in public health systems and teaching institutions [7]. In contrast, the simplified workflow presented here relies on conventional materials and analog techniques, making it more accessible and reproducible in a wide range of clinical settings.

A particularly novel feature of this technique is its potential to preserve the physiological primate space distal to the primary canine. In the primary dentition, primate spaces play a crucial biological role in accommodating the eruption of permanent incisors and canines, facilitating spontaneous anterior alignment and reducing the risk of crowding. Despite their importance, few studies have specifically evaluated whether space maintainers respect or disrupt these natural spacing patterns [1, 3]. Conventional soldered appliances often require rigid extensions toward the distal surface of the primary canine, which may inadvertently encroach upon or eliminate this physiological space [2]. In contrast, the present design, using a stock tooth prototype and adhesive fixation without rigid soldering, allows for a more anatomical and growth‐compatible adaptation. This feature may be clinically relevant, as preservation of primate spaces could reduce the need for future interceptive orthodontic procedures and promote more favorable eruption dynamics.

In recent years, digital workflows using CAD‐CAM and 3D‐printing technologies have been introduced for the fabrication of esthetic and functional space maintainers [8, 11, 12]. While these approaches offer excellent precision and customization, their widespread implementation remains limited by high equipment costs, need for specialized training, and restricted availability in many regions. From a global health perspective, simplified analog techniques continue to play an essential role in delivering effective pediatric dental care [4]. The present case demonstrates that it is possible to achieve satisfactory esthetic and functional outcomes using conventional materials, without reliance on advanced digital infrastructure.

The favorable clinical and radiographic outcomes observed at the 12‐month follow‐up, including stable occlusal contacts, maintenance of space, and normal development of the permanent successor, suggest that the proposed technique can fulfill its intended preventive and functional objectives. However, this report describes a single clinical case, which inherently limits the generalizability of the findings. Future studies with larger samples, longer follow‐up periods, and quantitative outcome measures are necessary to evaluate appliance longevity, periodontal response, influence on eruption patterns, and long‐term occlusal stability.

Additionally, clinicians should remain attentive to the dynamic nature of craniofacial growth during the mixed dentition period. Regular monitoring is essential to detect any deviations in eruption sequence, vertical dimension changes, or undesirable tooth movements. Space maintainers should not be viewed as static devices, but rather as part of a continuous interceptive strategy that adapts to the child's developmental needs.

## 4 Conclusions

The simplified technique presented in this case report represents a practical, low‐cost, and biologically oriented alternative for the fabrication of esthetic‐functional space maintainers in pediatric patients. By eliminating the need for orthodontic wire bending, silver or laser soldering, and CAD‐CAM technology, this approach facilitates clinical implementation while maintaining anatomical fidelity, restoring occlusal function, and preserving arch space during critical stages of dental development.

In addition to its technical simplicity, the proposed design emphasizes growth‐compatible principles, including respect for physiological spacing patterns and natural tooth morphology. These characteristics may contribute to more favorable eruption dynamics and occlusal development. Although further longitudinal studies with larger samples are required to confirm its long‐term effectiveness, this technique offers a promising and accessible option for preventive and interceptive orthodontic care.

## Author Contributions

**Luciana Duarte Caldas:** conceptualization, methodology, investigation, data curation, writing – original draft, writing – review and editing. **Anne Vitória de Jesus Ramos:** investigation, data curation, methodology, writing – original draft, writing – review and editing. **Luísa Schubach da Costa Barreto:** interpretation of data, writing – original draft, writing – review and editing. **Katharina Morant Holanda de Oliveira Vanderlei:** project administration, supervision, writing – original draft, writing – review and editing. All authors have read and approved the final manuscript.

## Funding

The authors have nothing to report.

## Ethics Statement

This case report did not require evaluation by an institutional ethics committee, as it describes the clinical management of a single pediatric patient without identifying information, in accordance with current ethical guidelines for case reports.

## Consent

Written informed consent for publication (including clinical data, radiographs, and intraoral/extraoral photographs) was obtained from the child's legal guardian prior to manuscript preparation. All procedures were conducted in accordance with the ethical principles outlined in the Declaration of Helsinki.

## Conflicts of Interest

The authors declare no conflicts of interest.

## Data Availability

The data supporting the findings of this study are fully presented within the article. Additional information is available from the first author [Luísa Schubach da Costa Barreto] upon reasonable request.
